# MITE*_Aba12_*, a Novel Mobile Miniature Inverted-Repeat Transposable Element Identified in *Acinetobacter baumannii* ATCC 17978 and Its Prevalence across the *Moraxellaceae* Family

**DOI:** 10.1128/mSphereDirect.00028-19

**Published:** 2019-02-20

**Authors:** Felise G. Adams, Melissa H. Brown

**Affiliations:** a College of Science and Engineering, Flinders University, Bedford Park, South Australia, Australia; University of Iowa; University of Sydney; Monash University

**Keywords:** *Acinetobacter*, genetic evolution, insertion sequences, nonautonomous elements, transposons

## Abstract

Insertion sequences (IS) are fundamental mediators of genome plasticity with the potential to generate phenotypic variation with significant evolutionary outcomes. Here, a recently active miniature inverted-repeat transposon element (MITE) was identified in a derivative of Acinetobacter baumannii ATCC 17978 after being subjected to stress conditions. Transposition of the novel element led to the disruption of the *hns* gene, resulting in a characteristic hypermotile phenotype. DNA identity shared between the terminal inverted repeats of this MITE and coresident IS*Aba12* elements, together with the generation of 9-bp target site duplications, provides strong evidence that IS*Aba12* elements were responsible for mobilization of the MITE (designated MITE*_Aba12_*) within this strain. A wider genome-level survey identified MITE*_Aba12_* in 30 additional *Acinetobacter* genomes at various frequencies and one Moraxella osloensis genome. Ninety MITE*_Aba12_* copies could be identified, of which 40% had target site duplications, indicating recent transposition events. Elements ranged between 111 and 114 bp; 90% were 113 bp in length. Using the MITE*_Aba12_* consensus sequence, putative outward-facing Escherichia coli σ70 promoter sequences in both orientations were identified. The identification of transcripts originating from the promoter in one direction supports the proposal that the element can influence neighboring host gene transcription. The location of MITE*_Aba12_* varied significantly between and within genomes, preferentially integrating into AT-rich regions. Additionally, a copy of MITE*_Aba12_* was identified in a novel 8.5-kb composite transposon, Tn*6645*, in the M. osloensis CCUG 350 chromosome. Overall, this study shows that MITE*_Aba12_* is the most abundant nonautonomous element currently found in *Acinetobacter*.

**IMPORTANCE** One of the most important weapons in the armory of *Acinetobacter* is its impressive genetic plasticity, facilitating rapid genetic mutations and rearrangements as well as integration of foreign determinants carried by mobile genetic elements. Of these, IS are considered one of the key forces shaping bacterial genomes and ultimately evolution. We report the identification of a novel nonautonomous IS-derived element present in multiple bacterial species from the *Moraxellaceae* family and its recent translocation into the *hns* locus in the A. baumannii ATCC 17978 genome. The latter finding adds new knowledge to only a limited number of documented examples of MITEs in the literature and underscores the plastic nature of the *hns* locus in A. baumannii. MITE*_Aba12_*, and its predicted parent(s), may be a source of substantial adaptive evolution within environmental and clinically relevant bacterial pathogens and, thus, have broad implications for niche-specific adaptation.

## Abstract (precis)

One of the most important weapons in the armory of *Acinetobacter* is its impressive genetic plasticity, facilitating rapid genetic mutations and rearrangements as well as integration of foreign determinants carried by mobile genetic elements. Of these, IS are considered one of the key forces shaping bacterial genomes and ultimately evolution. We report the identification of a novel nonautonomous IS-derived element present in multiple bacterial species from the *Moraxellaceae* family and its recent translocation into the *hns* locus in the A. baumannii ATCC 17978 genome. The latter finding adds new knowledge to only a limited number of documented examples of MITEs in the literature and underscores the plastic nature of the *hns* locus in A. baumannii. MITE*_Aba12_*, and its predicted parent(s), may be a source of substantial adaptive evolution within environmental and clinically relevant bacterial pathogens and, thus, have broad implications for niche-specific adaptation.

## INTRODUCTION

Acinetobacter baumannii has been classed as one of the most predominant pathogens responsible for multidrug-resistant (MDR) nosocomial infections worldwide (1). Aside from its notorious MDR phenotype, A. baumannii also displays a remarkable capacity to persist on a variety of inanimate surfaces for extended periods, providing a reservoir for infection and facilitating transmission throughout clinical settings (2, 3). Significant work has been undertaken to identify and track the arsenal of genes that contribute to the impressive persistence and resistance strategies available to A. baumannii (4–8). This has identified a highly dynamic and plastic genome, dominated by numerous integration events as well as alterations in expression of intrinsic genes modulated through mutations and deletion and/or insertion of mobile genetic elements (MGEs) (9–11). MGEs are present in nearly all prokaryote genomes and constitute the “mobilome,” a term which has gained significant traction in recent years, driven by the increase in infections caused by MDR isolates. The mobilome itself is comprised of a number of genetic entities, including plasmids, bacteriophages, gene cassettes in integrons, and transposable elements, all capable of capturing and disseminating genetic material across bacterial genomes via horizontal gene transfer (HGT) (12).

Of the above-mentioned entities, transposable elements are seen as a major contributor to niche-specific adaptive evolution. They are capable of moving from one position to another within a given genome and are often associated with the dissemination of antimicrobial resistance determinants (13–15). One of the simplest autonomous types of mobile elements is the insertion sequence (IS), consisting of a transposase gene(s) that is typically bordered by terminal inverted repeats (TIRs), designated left (IRL) and right (IRR) relative to the direction of the transposase gene. The TIRs contain multiple domains required for transposase binding, donor DNA cleavage, and strand transfer, supporting the integration of the elements into host DNA via replicative or nonreplicative mechanisms (16). As a consequence of insertion, short direct repeat sequences of the target DNA are often generated (target site duplications, or TSDs), which differ in length and degree of sequence specificity depending on the IS element being translocated (17). Movement of an IS to a new location within a genome offers a variety of possible integration sites. Although some IS display clear trends/preferences in target sites, the large majority of IS demonstrate low target specificity (18).

Small mobile elements can be further delineated based on their movement autonomy. A limited range of nonautonomous elements exist in bacteria, such as repetitive extragenic palindromic sequences, Tn*3*-derived inverted-repeat miniature elements (TIMEs), and miniature inverted-repeat transposable elements (MITEs) (19–21). Like eukaryotic MITEs (22), bacterial MITEs are small (∼50 to 600 bp) AT-rich sequences that have lost their cognate transposase gene and, thus, contain noncoding DNA that in most, but not all, cases are flanked by TIRs (23). Based on their origins, MITEs can be categorized as type I or type II and are generated by internal deletion of parent transposable elements or by random convergence of TIR sequences, respectively (24). Movement of these elements is thought to be mediated by transposases of a coresident parental element acting in *trans*. The site of integration and length of the TSDs of MITEs are generally identical or highly similar to that of the coresident IS parent (23). Since their identification in bacteria (25), a number of these elements have been documented from a diverse range of species, where many have significantly influenced the evolutionary tempo of their host genomes (20). These elements are often overlooked due to the absence of a recognizable coding sequence (CDS) and their tendency to reside in intergenic regions. Thus, they represent a largely unexplored field in microbial genomics.

Through characterization of a subset of morphologically distinct colonies isolated during desiccation stress analyses, we identified a novel MITE that transposed to a new location within the A. baumannii ATCC 17978 genome. Due to shared similarities in TIRs and TSD sequence length, the 113-bp sequence is predicted to have proliferated through the activity of the transposase encoded by resident IS*Aba12* elements present in ATCC 17978 and, thus, was named MITE*_Aba12_*. The prevalence of this novel, nonautonomous MGE across all publicly available sequenced bacterial genomes was examined and insights gained with respect to its transposition activity as well as its overall function and evolution.

## RESULTS

### Construction of *qseBC* and *ygiW* deletion derivatives in A. baumannii ATCC 17978.

The regulatory mechanisms that coordinate the expression of many A. baumannii virulence factors remain largely unknown. One regulatory mechanism employed by bacteria, including A. baumannii, is two-component signal transduction systems (TCS) (26). The TCS *qseBC* (ACX60_06100/05) and its upstream hypothesized target gene, which encodes the putative signal peptide *ygiW* (ACX60_06095), were deleted by allelic replacement in A. baumannii ATCC 17978 (GenBank accession no. CP012004.1), generating the Δ*qseBC* and Δ*ygiW* derivatives, respectively. To ensure the introduced mutations did not affect cell viability, growth curves assessing optical density at 600 nm (OD_600_) were undertaken in lysogeny broth (LB) medium and measured hourly over an 8-h period. No significant growth perturbations were identified for the Δ*qseBC* or Δ*ygiW* strain compared to growth of wild-type (WT) ATCC 17978 parent cells under the tested conditions (data not shown).

### Disruption of the *hns* gene after desiccation stress.

**FIG 1:**
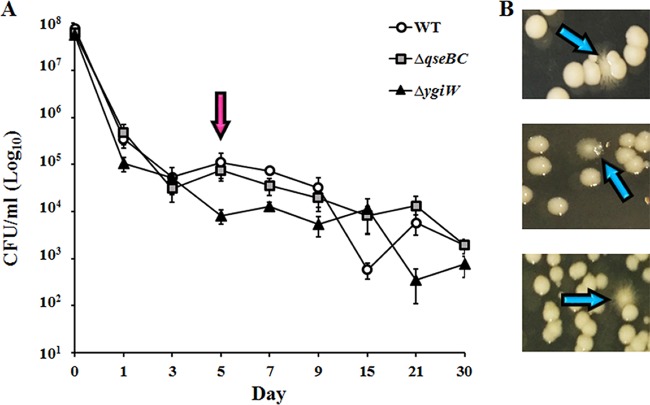
Identification of hypermotile variants from A. baumannii ATCC 17978 WT, Δ*qseBC*, and Δ*ygiW* strains after desiccation stress. (A) Desiccation survival was determined by enumeration of viable cells (CFU/ml) over a 30-day period. Markers represent mean values of viable cells and error bars the standard errors of the means calculated on days 0, 1, 3, 5, 7, 9, 15, 21, and 30. Four biological replicates were undertaken over two independent experiments. The pink arrow indicates the day that hypermotile variants were identified. (B) Images of hypermotile variants (blue arrows) obtained from rehydrated desiccated cells after ON incubation at 37°C on 1% LB agar.

To analyze the impact of deletion of the target genes in A. baumannii ATCC 17978, the constructed mutant strains were subjected to a number of *in vitro* assays, one of which was survival under desiccating conditions. No significant differences in survival compared to that of the WT were seen over the 30-day test period (Fig. 1A). However, on day 5 a subset of morphologically distinct colonies was identified during quantification of viable cells. These colonies displayed irregular edging reminiscent of a previously seen hypermotile phenotype (27) (Fig. 1B). In total, seven hypermotile isolates were identified, five from the Δ*ygiW* strain and one each from the Δ*qseBC* and WT backgrounds.

**FIG 2:**
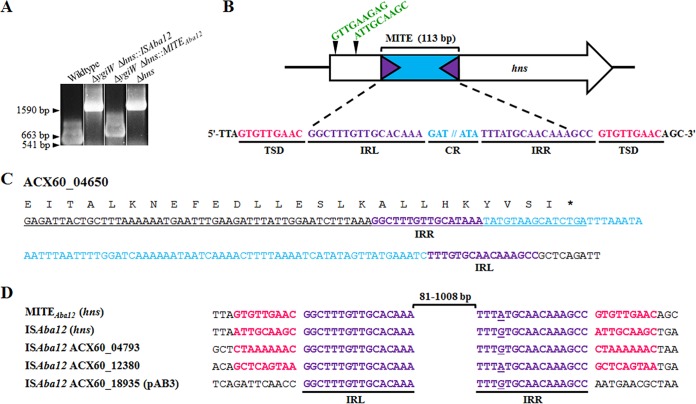
Insertions in the *hns* locus from hypermotile variants and relationship between IS*Aba12* and MITE*_Aba12_*. (A) Examples of amplicons generated from PCR across the *hns* locus from hypermotile isolates compared to the wild type and the previously identified Δ*hns* mutant (27). The amplicon from the Δ*ygiW* Δ*hns*::MITE*_Aba12_* strain (663 bp) was 122 bp larger than that from the wild-type control (541 bp), while the Δ*ygiW* Δ*hns*::IS*Aba12* strain yielded the same size product as the Δ*hns* control (1,590 bp). (B) The open white arrow depicts the *hns* gene (ACX60_16755) and direction of transcription, and black triangles with green nucleotide sequences represent the TSD for the two integration sites identified previously (29) as well as in this study. The 113-bp MITE is comprised of an 81-bp central region (CR; blue) flanked by 16-bp imperfect inverted repeat sequences (IRL and IRR; purple). //, break in DNA sequence. The novel insertion site/TSD sequences are in pink. The figure is not drawn to scale. (C) Location of MITE*_Aba12_* in the A. baumannii ATCC 17978 genome. The 3′ end of ACX60_04650 is fused to MITE*_Aba12_*, leading to a truncation and the formation of a pseudogene. The deduced amino acid sequence for the modified ACX60_04650 is designated by a single letter code above the underlined nucleotide sequence, and the asterisk indicates the proposed stop codon. Purple and blue nucleotides represent TIR and CR, respectively, of MITE*_Aba12_*. (D) Nucleotide alignment of 12 bp up- and downstream of the MITE*_Aba12_* element in *hns* of the A. baumannii ATCC 17978 Δ*ygiW* strain, IS*Aba12* elements present in ATCC 17978, and IS*Aba12* in *hns* [IS*Aba12* (*hns*)] (27). TIR and TSD are in purple and pink, respectively. Purple underlined nucleotides represent the mismatching base in IRR. The black bracket indicates the size of the region between IRL and IRR, either 81 bp for MITE*_Aba12_* or up to 1,008 bp for IS*Aba12*.

A previous study undertaken in A. baumannii ATCC 17978 showed that disruption of the histone-like nucleoid structuring (*hns*) gene by an IS (subsequently designated IS*Aba12* by ISfinder) (28) led to a number of phenotypic alterations, including hypermotility (27). Given the similarity in colony morphology between the set of hypermotile isolates identified after desiccation stress in this study and that previously seen for the Δ*hns* strain (29), our investigations initially focused on this global regulator. PCR amplifications across the *hns* loci of the hypermotile strains identified that all amplicons were larger than the WT control (Fig. 2A). DNA sequencing of these products revealed insertion of IS*Aba12* in three cases, originating from each of the three different background strains, which were located in two previously identified integration sites (29) (Fig. 2B). In the remaining four strains, all based on the Δ*ygiW* background, a shorter insertion in *hns* was detected, and sequencing of one example revealed a 113-bp element integrated into a novel site (Fig. 2B). To determine if the integrated element was stably inserted in *hns* of the Δ*ygiW* strain, PCR screening after five consecutive passages in liquid culture from five biological replicates was undertaken. All samples maintained the element within *hns* (data not shown). To examine whether isolates with a disrupted *hns*, irrespective of the site/type of integration, still produced the distinctive hypermotile phenotype, their motility phenotypes were assessed and found to be comparable to that seen for the previously identified *hns* mutant derivative (27, 30) (see Fig. S1 in the supplemental material). Complementation with a WT copy of *hns* (ACX60_16755) carried on the pWH1266 shuttle vector (27) restored all isolates to their parental nonmotile phenotype (Fig. S1).

10.1128/mSphereDirect.00028-19.1 FIG S1 Motility of A. baumannii ATCC 17978 variants and complemented derivatives. Cells grown overnight were used as the inoculum for motility assays on LB medium containing 0.25% agar. WT ATCC 17978, Δ*qseBC*, and Δ*ygiW* cells displayed a nonmotile phenotype. Derivatives of these strains with an *hns* gene interrupted by IS*Aba12* or MITE*_Aba12 _*and Δ*hns* (27) displayed a hypermotile phenotype, dispersing from the original inoculum site to cover the entire plate surface. Reintroduction of a WT copy of *hns* on the shuttle vector pWH1266 (pWH0268) returned strains to the parental nonmotile phenotype. Images are a representative example of results obtained. Download FIG S1, TIF file, 1.9 MB. Copyright © 2019 Adams and Brown. 2019 Adams and Brown This content is distributed under the terms of the Creative Commons Attribution 4.0 International license.

### Identification and characterization of a novel active MITE in A. baumannii ATCC 17978.

To characterize this novel 113-bp element found in the A. baumannii Δ*ygiW* strain, its DNA sequence and that of its insertion site in *hns* were analyzed. This revealed that the 113-bp element carried 16-bp imperfect TIR sequences (different in 1 nucleotide) and an 81-bp core region and generated 9-bp TSDs on insertion into *hns* (Fig. 2). The element is AT rich (78%) and does not contain any known CDS (31). Taken together, these traits strongly suggested that this element is a MITE (23).

To identify the abundance of the MITE within the A. baumannii ATCC 17978 genome, the 113-bp MITE sequence in *hns* from the Δ*ygiW* background was used as a query for BLASTN searches. Only one copy at the 3′ end of the ACX60_04650 locus, encoding a hypothetical protein harboring a partial KAP-family NTPase motif (32), was identified, fusing this gene with 31 bases from the MITE (Fig. 2C) and generating a premature stop codon. Comparative analyses with A. baumannii D36 revealed that the protein was 398 amino acids shorter and is therefore likely to be nonfunctional (data not shown). Genes coding for KAP NTPases are known to be frequently disrupted, leading to pseudogene formation (33). PCR with primers specific for the ACX60_04650 location (see Table 4) identified that the MITE was maintained in this position in the Δ*ygiW* Δ*hns*::MITE strain. Consequently, there are two MITE copies in the Δ*ygiW* background, one at the ACX60_04650 locus and an additional copy located in the *hns* gene, inferring duplication of the novel element (data not shown).

### IS*Aba12* is the proposed autonomous parent of the novel MITE in A. baumannii ATCC 17978.

To identify the potential parent element that may have aided in translocation of the MITE, IS present in the ATCC 17978 chromosome and pAB3 plasmid (GenBank accession numbers CP012004.1 and CP012005.1, respectively) were first identified from results generated by ISseeker (11). Subsequent manual examination of the length and sequence of their TIRs and TSDs revealed that IS*Aba12* provided the best match to those of the MITE. IS*Aba12* harbors a single open reading frame coding for a transposase, with a characteristic DDE catalytic motif, between its 16-bp TIRs and generates 9-bp TSDs upon insertion (28). Thus, the novel MITE was most likely translocated into *hns* by a coresident IS*Aba12* transposase present in ATCC 17978 and will be referred to as MITE*_Aba12_*.

As MITE*_Aba12_* does not contain a transposase gene, it is not possible to define IRL and IRR sequences relative to this gene. Two of the three copies of IS*Aba12* in ATCC 17978 (at loci ACX60_04795 and ACX60_18935) have identical TIRs that perfectly match one TIR of MITE*_Aba12_*. However, the nonidentical TIRs of the third copy of IS*Aba12* (ACX60_12380) each perfectly match one TIR of MITE*_Aba12_*, allowing IRL and IRR of MITE*_Aba12_* to be designated relative to this IS.

### MITE*_Aba12_* is present in a diverse range of species from the *Moraxellaceae* family.

To identify whether MITE*_Aba12_* is widespread or restricted to A. baumannii ATCC 17978, the sequence found within *hns* from the Δ*ygiW* Δ*hns*::MITE*_Aba12_* strain was used as a query to search bacterial genomes present in publicly available databases (10 July 2018). Orthologs of MITE*_Aba12_* were identified in both chromosomes and plasmids, with an additional 30 strains from the *Acinetobacter* genus and one from Moraxella osloensis harboring the element at various frequencies (Table 1). MITE*_Aba12_* was found within a range of environmental *Acinetobacter* species, with the greatest number of copies identified (*n *=* *22) in A. baumannii DS002, isolated from soil in Anantapur, India, in 2005. A number of *Acinetobacter* strains isolated from patients and hospital sewage in multiple countries also carried copies of MITE*_Aba12_*, inferring its presence and dissemination into clinically relevant isolates worldwide. *Acinetobacter* sp. strains ACNIH2, SWBY1, and WCHA45 and A. johnsonnii XBB1 possessed MITE*_Aba12_* on the chromosome as well as in plasmids (Table 1). An additional number of plasmid sequences carrying MITE*_Aba12_* were also identified (Table 1), but their corresponding chromosome sequences are not available. Copies of MITE*_Aba12_* identified in the A. baumannii PR07 genome (GenBank accession number CP012035.1) were not included in further analyses as the genome contained strings of undetermined bases and was not of a high enough quality.

**TABLE 1:** Bacterial strains that harbor full-length MITE*_Aba12_* elements

Strain or plasmid	No. of MITE*_Aba12_* elements per strain	Isolation source/origin	Accession no. and reference or source
Strain			
A. baumannii DS002	22	Soil, India	CP027704.1, unpublished
A. indicus SGAir0564	10	Air, Singapore	CP024620.1 (75)
A. johnsonii XBB1^*a*^	7	Hospital sewage, USA	CP010350.1 (76)
A. junii 65	5	Limnetic water, Russia	CP019041 (77)
* Acinetobacter* sp. strain SWBY1^*a*^	5	Hospital sewage, China	CP026616.1, unpublished
A. baumannii B8300	4	Human bloodstream, southern India	LFYY00000000.1 (78)
* Acinetobacter* sp. strain ACNIH1	3	Hospital plumbing, USA	CP026420.1 (79)
A. baumannii ABNIH28	3	Hospital plumbing, USA	CP026125 (79)
* Acinetobacter* sp. strain TGL-Y2	2	Frozen soil, China	CP015110.1, unpublished
A. baumannii B8342	2	Human bloodstream, southern India	LFYZ00000000.1, (80)
M. osloensis CCUG 350	1	Human cerebrospinal fluid, USA	CP014234.1, unpublished
A. haemolyticus TJS01	1	Human respiratory tract, China	CP018871.1, unpublished
* Acinetobacter* sp. strain NCu2D-2	1	Murine trachea, Germany	CP015594 (81)
* Acinetobacter* sp. strain ACNIH2^*a*^	1	Hospital plumbing, USA	CP026412.1 (79)
A. baumannii ATCC 17978	1	Human meninges, France	CP012004.1 (5)
A. junii WCHAJ59	1	Hospital sewage, China	CP028800.1, unpublished
A. baumannii AR_0083	1	Unknown	CP027528.1, unpublished
* Acinetobacter* sp. strain WCHA45^*a*^	1	Sewage, China	CP028561.1, unpublished
A. baumannii MAD^*b*^	1	Human skin, France	AY665723.1 (82)
			
Plasmids			
A. schindleri SGAir0122, pSGAir0122	2	Air, Singapore	CP025619.1 (83)
A. baumannii A297 (RUH875), pA297-3	1	Human urinary tract, Netherlands	KU744946 (46)
A. johnsonnii XBB1, pXBB1-9	1	Hospital sewage, USA	CP010351.1 (76)
A. lwoffii ED45-23, pALWED2.1	1	Permafrost, Russia	KX426229 (53)
A. baumannii AbPK1, pAbPK1a	1	Ovine respiratory tract, Pakistan	CP024577 (84)
* Acinetobacter* sp. strain DUT-2, unnamed 1	1	Marine sediment, China	CP014652, unpublished
* Acinetobacter* sp. strain BW3, pKLH207	1	Stream water, USA	AJ486856 (85)
A. towneri strain G165, pNDM-GJ01	1	Human stool, China	KT965092 (86)
A. baumannii D46, pD46-4	1	Human urine, Australia	MF399199 (52)
* Acinetobacter* sp. strain ACNIH2, pACl-3569	1	Hospital plumbing, USA	CP026416.1 (79)
* Acinetobacter* sp. strain WCHA45, pNDM1_100045	1	Hospital sewage, China	CP028560.1, unpublished
A. baumannii CHI-32, pNDM-32	1	Human bloodstream, India	LN833432.1, unpublished
A. defluvii WCHA30, pOXA58_010030	1	Hospital sewage, China	CP029396.1, unpublished
A. pittii WCHAP005069, pOXA58_005069	1	Clinical isolate, China	CP026086.1, unpublished
A. pittii WCHAP100004, pOXA58_100004	1	Clinical isolate, China	CP027249.1, unpublished
A. pittii WCHAP005046, pOXA58_005046	1	Clinical isolate, China	CP028573.1, unpublished
* Acinetobacter* sp. strain SWBY1, pSWBY1	1	Hospital sewage, China	CP026617.1, unpublished

a Strains where MITE*_Aba12_* is present on both chromosomal and plasmid DNA.

b In A. baumannii MAD, MITE*_Aba12_* was found on a 7.8-kb stretch of sequenced DNA rather than a full-length chromosome (82).

Using ISseeker (11), it was found that approximately 18.5% of the 1,035 A. baumannii genomes examined harbored at least one copy of IS*Aba12*, with an average of 5.6 copies per genome (data not shown). Using the ISfinder tool (28), four relatives of IS*Aba12* were identified: IS*Aba13*, IS*Alw1*, IS*Aha1,* and IS*Aha2* (Table 2). These elements are present at various frequencies in *Acinetobacter* genomes, and the transposases encoded within the elements share between 92 and 94% amino acid identity with the transposase in IS*Aba12*. Importantly, they have the same perfect 16-bp TIR sequence as the majority of IS*Aba12* elements (Table 2). This led us to investigate whether other characterized IS contain TIR sequences similar to those in MITE*_Aba12_* and thereby could translocate the nonautonomous element. An additional nine IS elements were found to have TIR sequences similar or identical to those of MITE*_Aba12_* (Table 2). These IS elements are of a length similar to that of IS*Aba12*, ranging from 1,023 to 1,052 bp, with the majority also generating 9-bp TSDs (Table 2). Comparisons of MITE*_Aba12_* against the sequences of each IS listed in Table 2 revealed nucleotide identity was confined to only the TIR sequences; thus, the origins of MITE*_Aba12_* from an IS could not be readily deduced (data not shown).

**TABLE 2:** IS with TIR closely related to those of IS*Aba12* and MITE*_Aba12_*

IS name^*a*^	IRL sequence	IRR sequence	Length (bp)	TSD (bp)
MITE*_Aba12_*	GGCTTTGTTGCACAAA	GGCTTTGTTGCATAAA	113	9
IS*Aba12*	GGCTTTGTTGCACAAA	GGCTTTGTTGCACAAA	1,039	9
IS*17*	GGCTTTGTTGCACAAA	GGCTTTGTTGCACAAA	1,040	9
IS*Aba5*^*b*^	GGCTTTGTTGCACAAA	GGCTTTGTTGCATAAA	1,044	ND
IS*Aba7*	GGCTTTGTTGCATAAA	GGCTTTGTTGCACAAA	1,039	9
IS*Aba10*	GGCTTTGTTGCATAAATA	GGCTTTGTTGCACAAATA	1,023	9
IS*Aba13*	GGCTTTGTTGCACAAA	GGCTTTGTTGCACAAA	1,039	9
IS*Aba40*	GGCTTTGTTGCACAAA	GGCTTTGTTGCACAAA	1,039	9
IS*Aha1*	GGCTTTGTTGCACAAAC	GGCTTTGTTGCACAAAC	1,039	4
IS*Aha2*	GGCTTTGTTGCACAAA	GGCTTTGTTGCACAAA	1,040	ND
IS*Aha3*	GGCTTTGTTGCACAAA	GGCTTTGTTGCATAAA	1,039	ND
IS*Ajo1*	GGCTTTGTTGCACAAA	GGCTTTGTTGCATAAA	1,039	3
IS*Alw1*	GGCTTTGTTGCACAAAG	GGCTTTGTTGCACAAAG	1,038	ND
IS*Ecl7*	GGCTTTGTTGCACAAA	GGCTTTGTTGCATAAA	1,052	9
IS*Nov2*	GGCTTTGTTGCGCAAAT	GGCTTTGTTGCATAAAT	1,048	9

a Abbreviations: IS, insertion sequence; IRL, inverted repeat left; IRR, inverted repeat right; TSD, target site duplication; ND, not determined.

b The transposase of IS*Aba5* is thought to be inactive (28).

### MITE*_Aba12_* is a highly conserved mobile element with potential to affect expression of neighboring host genes.

**FIG 3:**
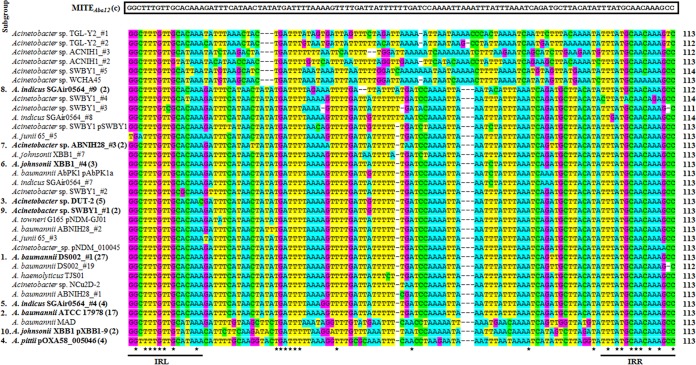
Nucleotide alignment of all MITE*_Aba12_* elements identified in this study. The nucleotide sequence above the alignment (black box) denotes the consensus sequence, MITE*_Aba12_*(c), derived using WebLogo software (35). MITE*_Aba12_* sequences with nucleotide variations are displayed. Subgroup representatives are numbered and in boldface type with numbers in parentheses indicating the total number of MITE*_Aba12_* copies with that sequence. A, T, G, and C nucleotides are denoted in blue, yellow, purple, and green boxes, respectively. Black lines and asterisks represent the terminal inverted repeats (IRL and IRR) and conserved bases, respectively. See Table S1 for a full list of MITE*_Aba12_* elements included in each subgroup and Table 1 for strain accession numbers.

To examine sequence identity across all the identified MITE*_Aba12_* copies, a multiple-sequence alignment using Clustal Omega (34) was performed. From the analysis of 90 MITE*_Aba12_* copies it was found that 10% of MITE*_Aba12_* copies diverged from the 113-bp consensus (Fig. 3). Three of the 10 MITE*_Aba12_* copies present in Acinetobacter indicus SGAir0564 are atypical; two were 112 bp, sharing 100% identity with each other, while the other is 114 bp. Similarly, in *Acinetobacter* sp. strain SWBY1, three of the five MITE*_Aba12_* copies differed from the consensus length, which included the smallest identified element at 111 bp and two at 114 bp. A further three atypical 112-bp MITE*_Aba12_* sequences were identified in the genomes of A. baumannii DS002 and *Acinetobacter* sp. strains TGL-Y2 and ACNIH1 (Fig. 3). Thus, a total of 34 different MITE*_Aba12_* sequences were identified, leading to the assignment of 10 subgroups. MITE*_Aba12_* sequence arrangements that harbored two copies or more were segregated into subgroups that were ordered from 1 to 10 based on the most to least abundant (Fig. 3 and Table S1). Significant variation existed across the central region of the element, as only 10 bases were conserved across all 90 identified copies (Fig. 3) (it is possible that some nucleotide differences identified across MITE*_Aba12_* copies can be attributed to sequencing errors). The MITE*_Aba12_* TIR sequences were the most conserved, as eight and nine of the 16-bp IRL and IRR sequences, respectively, were identical across all MITE*_Aba12_* copies analyzed (Fig. 3). Overall, no preference in the orientation of MITE*_Aba12_* in the genomes could be identified (data not shown).

10.1128/mSphereDirect.00028-19.3 TABLE S1 List of MITE*_Aba12_* elements and their corresponding strain names that formulate MITE*_Aba12_* subgroups 1 to 10. Download Table S1, DOCX file, 0.01 MB. Copyright © 2019 Adams and Brown. 2019 Adams and Brown This content is distributed under the terms of the Creative Commons Attribution 4.0 International license.

**FIG 4:**
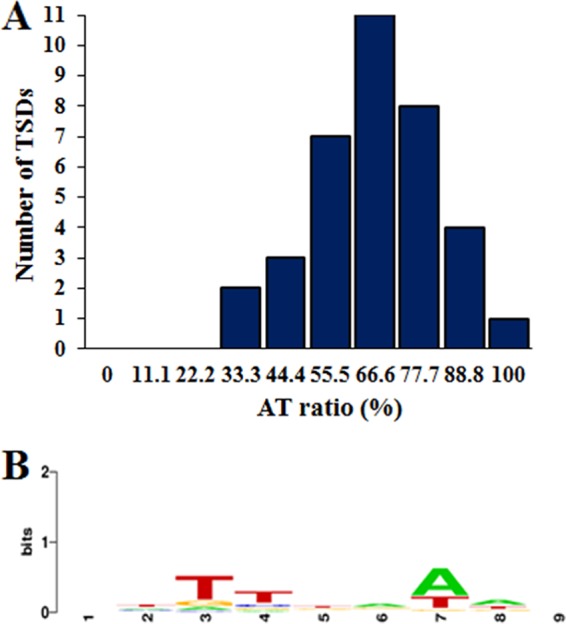
Characterization of target site duplications flanking MITE*_Aba12_*. (A) Graphical representation of AT richness (%) identified from all target site duplications flanking MITE*_Aba12_* elements. (B) Nucleotide logo generated from all target site duplication events using WebLogo software (35).

MITEs generally insert into AT-rich regions (23). Of the 90 identified MITE*_Aba12_* copies, 36 from 13 different genomes had 9-bp TSDs. From the 22 MITE*_Aba12_* copies in A. baumannii DS002, TSDs could be identified for 17, which could infer a burst of recent activity. Interestingly, all MITE*_Aba12_* elements from subgroup 4 were found in the same site and flanked by identical TSDs of 8 rather than 9 bp (TTTTTGTT). These elements were on large plasmids (∼62 to 112.5 kb), and BLASTN analyses using the MITE*_Aba12_* element together with ∼700 bp left and right of the sequence from pNDM-32 of A. baumannii CHI-32 identified they were all located in an identical position, sharing 100% identity across this ∼1.5-kb region (data not shown). Overall, MITE*_Aba12_* appeared to favor insertion into sequences with an AT ratio of ≥55.5%, as demonstrated by the skewed distribution of the columns to the right (Fig. 4A), although no identifiable trends in nucleotide sequence arrangements could be identified (Fig. 4B).

To assess how MITE*_Aba12_* could influence host gene expression, a consensus sequence, MITE*_Aba12_*(c), was generated using WebLogo (35) from all 113-bp MITE*_Aba12_* elements (*n *=* *81) (Fig. 3). At least two stop codons in all six reading frames can be identified after translation of the MITE*_Aba12_*(c) DNA sequence. Start codons followed by three, seven, or eight amino acids at the terminal ends of the element were identified in three of the reading frames (data not shown). Depending on the integration site in a given genome, these characteristics could allow for fusion with neighboring CDSs. Mfold (36) predicted weak secondary structures with Δ*G* of −23.99 or −26.94 kcal/mol in the two orientations of MITE*_Aba12_*(c) (IRL to IRR or IRR to IRL, respectively) (data not shown). Using the ARNold tool (37), no predicted Rho-independent transcriptional terminators were identified. The Softberry program BPROM predicted two outward-facing promoter sequences based on the −35 and −10 Escherichia coli σ70 promoter consensus sequences (Fig. S2). These sequences were also compared with the strong outward-facing promoter found in IS*Aba1* coupled with flanking sequence associated with overexpression of the *bla_ampC_* gene in A. baumannii CLA-1 (38) (Fig. S2B). To verify whether the two putative outward-facing promoters identified within MITE*_Aba12_*(c) could drive the production of mRNA transcripts, three previously published A. baumannii ATCC 17978-derived RNA-sequencing transcriptomes (30, 39) were aligned to the reference ATCC 17978 genome (GenBank accession number CP012004.1) using the Integrative Genomics Viewer program (40). Transcripts originating within the MITE*_Aba12_* sequence that could be attributed to the P*_out_* IRR putative promoter were identified across all three transcriptomes. However, transcripts reading out from P*_out_* IRL were limited (data not shown). Thus, it appears in ATCC 17978 that the P*_out_* IRR putative promoter within MITE*_Aba12_*(c) has the potential to influence host gene transcription.

10.1128/mSphereDirect.00028-19.2 FIG S2 Putative σ70 promoter sequences identified in MITE*_Aba12_*(c). The σ70 promoter sequences were predicted using the Softberry BPROM tool. (A) Pink and blue nucleotide sequences represent outward-facing promoters, reading out through the IRL and IRR sequences (*P*_out_ IRL and *P*_out_ IRR, respectively), and the orientation of transcription is shown by arrows. Nucleotides underlined and double underlined denote −10 box and −35 box sequences, respectively. Purple nucleotides denote IRL and IRR, with putative translational start codons in boldface with their corresponding putative ribosome binding sites (RBS) shaded in pink and blue, respectively. (B) Alignment of the putative *P*_out_ IRL and *P*_out_ IRR and RBSs in MITE*_Aba12_*(c) against the E. coli σ70 promoter and RBS consensus and *P*_out_ of IS*Aba1* coupled with the adjacent region and RBS in the IS*Aba1-*activated *bla*_ampC_ gene of A. baumannii CLA-1 (38). The nucleotide length between the −10 and −35 boxes [Sep. (bp)] is indicated. Download FIG S2, TIF file, 1.5 MB. Copyright © 2019 Adams and Brown. 2019 Adams and Brown This content is distributed under the terms of the Creative Commons Attribution 4.0 International license.

The fusion of small mobile elements with neighboring genes can affect gene function and in some cases lead to improved host fitness or formation of new proteins (41, 42). The exhaustive analysis conducted on MITE*_Aba12_* in publicly available GenBank entries revealed that some insertions of MITE*_Aba12_* interrupted host genes, and in some cases the encoded protein could be fused with up to 19 amino acids encoded by MITE*_Aba12_* sequences (data not shown). MITE*_Aba12_* elements located in pAbPK1a from A. baumannii AbPK1 and in the chromosomes of A. baumannii B8300 and *Acinetobacter* sp. strain ACNIH1_#2 could create fusions to the 5′ end of adjacent genes. Each had incorporated nucleotides reading outwards from the TIR of MITE*_Aba12_* to generate the first four amino acids (MQQS) of the neighboring CDS. These particular arrangements also placed the host gene in proximal distance to the P*_out_* IRR promoter sequences, and given its activity in ATCC 17978, the element could also influence the expression of fused genes.

### MITE*_Aba12_* in *M. osloensis* CCUG 350 is located within a novel composite transposon.

**FIG 5:**
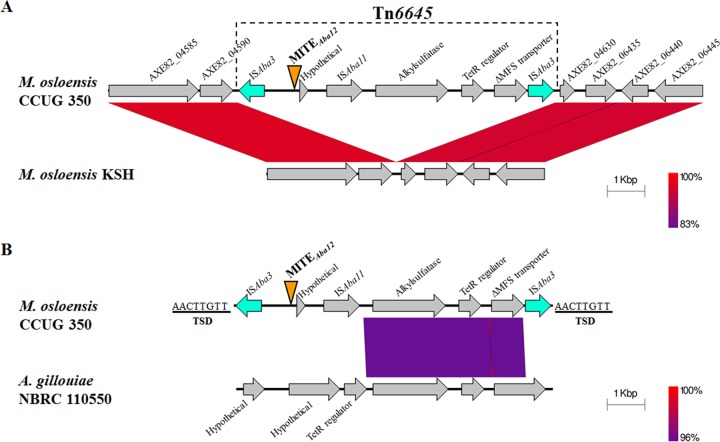
MITE*_Aba12_* is located within Tn*6645* in Moraexella osloensis CCUG 350. Gray arrows indicate the direction of transcription, and blue arrows represent IS*Aba3* elements forming the boundaries of Tn*6645*. Identity between regions is indicated by the color gradient. (A) Alignment of nucleotide sequence from AXE82_04585 to AXE82_06445 in M. osloensis CCUG 350 and the corresponding region in strain KSH (73) (GenBank accession numbers CP014234.1 and CP024180.2, respectively). Gene names and locus tags are derived from M. osloensis CCUG 350 annotation. (B) Alignment of Tn*6645* from M. osloensis CCUG 350 and part of the A. guillouiae NBRC 110550 chromosome (GenBank accession number AP014630.1 [43]). Identity between Tn*6645* and A. guillouiae NBRC 110550 starts 80 bp downstream from the TIR of IS*Aba11*. The 8-bp TSDs flanking Tn*6645* are shown. The location of MITE*_Aba12_* is indicated by the orange triangle. Sequences were obtained from the NCBI database and aligned and visualized using the Easyfig 2.2.2 tool (74).

As previously stated, M. osloensis CCUG 350 carries one copy of MITE*_Aba12_* (Table 1). It lies within an 8.5-kb region absent from five closely related *M. osloensis* genome sequences (Fig. 5A shows the sequence alignment). IS were found at the terminal ends of the novel insert and shared highest identity with the IS*1* family member IS*Aba*3 (81% identity; E value, 5e−55) (28). Both terminal IS carried 24-bp TIR sequences (5′-GGTGGTGTTTCAAAAAGTATGCTG-3′), and TSDs of 8 bp were identified at each end of the 8.5-kb insert (Fig. 5B). These features make this sequence synonymous with a composite transposon (17) now named Tn*6645*. In M. osloensis CCUG 350, the MITE*_Aba12_* element was located between the IS*Aba*3 element and a gene of unknown function containing a DUF 2789 motif (E value, 4.2e−27) (32). Additionally, an IS*Aba11*-like element, an alkylsulfatase gene, a TetR-family transcriptional regulator gene, and a partial gene encoding a major facilitator superfamily transporter were identified within the composite transposon (Fig. 5). The insertion of IS*Aba*3 truncated the 3′ end of the transporter gene by 540 bp and therefore is likely nonfunctional (a pseudogene).

BLASTN searches were used to search for Tn*6645* in other bacterial genomes, but no additional full-length copies were identified (data not shown). However, approximately 4.3 kb of the 8.5-kb sequence aligned (96% identity) to a region in the chromosome of Acinetobacter guillouiae NBRC 110550 (43) (Fig. 5B). This region harbored the alkylsulfatase, TetR-family regulator, and the truncated transporter genes and may represent a source for this portion of the Tn*6645* cargo.

## DISCUSSION

Since their identification in bacteria 30 years ago, MITEs have been reported in a multitude of species, displaying significant diversity in their nucleotide sequence and functional properties (44). In this study, a novel MITE was identified in environmental and clinical isolates of *Acinetobacter* species, including A. baumannii, one of the leading bacterial organisms threatening human health (2). This novel element lacked any CDS that could produce a functional transposase, inferring that like other MITEs, MITE*_Aba12_* is activated in *trans*. Given the high similarity between the TIR sequences of MITE*_Aba12_* and those of IS*Aba12* (Fig. 2D), we propose the transposase from IS*Aba12* elements were responsible for MITE*_Aba12_* mobilization in the A. baumannii Δ*ygiW* strain. Whether IS*Aba12,* or another IS with a TIR similar to that of MITE*_Aba12_* (Table 2), can mobilize MITE*_Aba12_* will need to be experimentally examined.

With the addition of MITE*_Aba12_*, the list of nonautonomous elements reported in *Acinetobacter* grows to three. Like most prokaryotic MITEs, the two previously characterized MITEs from *Acinetobacter* isolates are flanked by TIRs and generate TSDs upon insertion (45, 46). Compared to MITE*_Aba12_*, both elements have been identified only on plasmid sequences and are approximately four times larger in size (439 and 502 bp, respectively) (45, 46). Identical copies of the MITE originally identified in *Acinetobacter* sp. strain NFM2 flank class 1 integrons carrying different resistance determinants in a number of *Acinetobacter* strains, forming a structure comparable to that of a composite transposon (45, 47–51). MITE-297 is found on the large conjugative plasmid pA297-3 present in the A. baumannii global clone 1 reference strain A297 (RUH875) (46). In pA297-3, two copies of MITE-297 flank a 76-kb region carrying numerous IS and a *mer* module which confers resistance to mercury (46). Interestingly, within pA297-3, MITE*_Aba12_* is also present in the intergenic region between the *merD* and 5-hydroxyisourate hydrolase precursor genes (data not shown). MITE*_Aba12_* is also found in an identical position in the ∼208-kb pD46-4 plasmid from A. baumannii D46 (52) and a 141-kb plasmid from *Acinetobacter* sp. strain DUT-2. Given the position of the element within these plasmids, we suggest that MITE*_Aba12_* has travelled with this *mer* operon, which may partly explain its distribution throughout these bacterial genomes. Our analyses also identified a copy of MITE*_Aba12_* flanked by two IS on the large nonconjugative plasmid pALWED2.1 from the Acinetobacter lwoffii strain ED45-23, isolated from uncontaminated Russian permafrost sediments dated to be 20,000 to 40,000 years old (53). To our knowledge, this is the most primitive strain that has been sequenced and shown to carry a copy of MITE*_Aba12_*. Interestingly, heavy-metal resistance operons identified on pALWED2.1 share identity with sequences from two additional *Acinetobacter* strains that also carry copies of MITE*_Aba12_* (53). Our data, which provide another example of MITE*_Aba12_* hitchhiking alongside resistance genes, supports the idea that HGT has played an important role in the evolution of heavy-metal resistance to confer a selective advantage to the organism (53).

Bacterial MITEs can possess various motifs that affect their own regulation and/or modulate expression of other genes within the residing genome (54–56). Using the MITE*_Aba12_*(c) consensus sequence identified as part of this study, putative outward-facing E. coli σ70 promoters could be identified in both orientations (see Fig. S2 in the supplemental material). IS*Aba1* is present in high copy numbers across a number of A. baumannii genomes and has been shown to have a significant impact on host gene expression and genome architecture (11). Additionally, IS*Aba1* is frequently implicated in increased antibiotic resistance, achieved by insertion upstream of resistance genes, namely, those encoding cephalosporinases or carbapenamases (38, 57, 58). Despite the putative promoter sequences not being maintained across all MITE*_Aba12_* elements, the two subgroups which exhibited the greatest number of conserved arrangements (subgroups 1 and 2, with 27 and 17 elements, respectively) have promoter sequences that exactly match the MITE*_Aba12_*(c) consensus (Fig. 3). Elements within these subgroups were derived from a variety of species from the *Moraxellaceae* family and isolated from geographically distinct locations (Table 1), suggesting that a selective pressure to maintain these nucleotides exists.

Analysis of the 90 MITE*_Aba12_* copies revealed that sequence conservation was mainly confined to their TIRs, and they only deviated in length from the MITE*_Aba12_*(c) consensus by a maximum of two nucleotides (Fig. 3). This is in contrast to significant size differences seen between variants of other types of bacterial MITEs (55, 59, 60). However, the lack of significant divergence seen within MITE*_Aba12_* copies indicates that the element was generated from a single event and dispersed through bacterial genomes via HGT.

Shared identity of IS*Aba12* and the additional 13 IS harboring TIRs similar to those of MITE*_Aba12_* was restricted to the TIR sequences (Table 2). Nevertheless, this finding significantly broadens the range of potential parental IS that could be capable of translocating MITE*_Aba12_*. However, further experimental evidence is required to confirm whether these IS can translocate MITE*_Aba12_*.

The observation of a MITE translocation within a prokaryote genome in real time is generally considered to be a rare event, as only a few examples have been documented in the literature (44). Remarkably, four separate instances where MITE*_Aba12_* underwent translocation into *hns*, all of which were observed within the A. baumannii Δ*ygiW* ATCC 17978 background, were identified. YgiW is known as a stress-induced protein in many Gram-negative bacterial species (61–64). For instance, in Salmonella enterica serovar Typhimurium, YgiW (renamed VisP, for virulence-induced stress protein) was shown to be critical in stress resistance *in vitro* and in virulence (64). Similar to that of S. enterica and other bacteria, the *ygiW* homolog found in A. baumannii also contains the characteristic bacterial oligonucleotide/oligosaccharide-binding fold domain (DUF388) (65) and is located immediately upstream of the *qseBC* TCS genes (data not shown). As transposition of IS is strongly controlled, most likely to reduce potential deleterious effects within the cell, we question whether the deletion of a protein involved in the stress response influenced the transposition and/or properties that regulate expression and subsequent movement of IS*Aba12*/MITE*_Aba12_* elements within the ATCC 17978 genome. Furthermore, as isolates displaying hypermotility were only identified once during desiccation analyses, we speculate that these events represent a transposition burst (66). This new phenomenon offers a substitute for the selfish DNA hypothesis, where these intermittent bursts of IS transposition can increase copy numbers and therefore assist in their maintenance within bacterial genomes.

H-NS is defined as a DNA architectural protein known to play multiple fundamental roles across a number of Gram-negative pathogens, including regulation of AT-rich horizontally acquired genes, many of which are involved in multiple stress responses (67, 68). Two distinct locations for IS*Aba12* insertions in the *hns* locus were previously identified in A. baumannii (27, 29). These were also the target sites for the IS*Aba12* insertions in this study, inferring these sequences are favored integration hotspots. MITE*_Aba12_* inserted into a novel location within *hns*, 151 bp from the start codon and upstream of the characterized DNA-binding domain (27). Two additional examples of IS-mediated disruption of *hns* in A. baumannii have been recently identified (9, 69). IS*Aba12*5 was shown to be responsible in both studies, integrating into the intergenic region downstream of *hns* (ACICU_00289). In one case, the last two amino acids of H-NS are altered and the protein extended for an additional three amino acids by the integration of complete and partial copies of the IS*Aba12*5 element (69). Collectively, these results infer that H-NS is a hot spot for disruption in A. baumannii, where a number of different integration sites have now been identified.

Transposable elements are a key driving force in the worrying increase in MDR isolates across many bacterial species, particularly within the *Acinetobacter* genus. Despite their small size, MITEs have been shown to be a significant contributor to genetic variation in a number of pathogens. In conclusion, this work has identified and characterized a new MGE, MITE*_Aba12_*, and determined its prevalence across the *Moraexellaceae* family. This also led to the identification of a novel composite transposon in M. osloensis CCUG 350, Tn*6645*. Due to the relatively small number of MITE*_Aba12_* copies identified in sequenced genomes, the element may be maintained neutrally or under tight regulatory control from a yet-to-be-identified host and/or environmental factor(s). The full effects of MITE*_Aba12_* on genetic variation and, thus, evolution of bacterial genomes, in addition to transcriptional and translational influences, have yet to be experimentally examined, opening a new and exciting avenue of research. The overall findings of this study not only illustrate the fluidity of the *Acinetobacter* pangenome but also highlight the importance of mobile sequences as vehicles for niche-specific adaptive evolution in a number of clinically and environmentally relevant bacterial pathogens.

## MATERIALS AND METHODS

### Bacterial strains, plasmids, media, and growth conditions.

A. baumannii ATCC 17978 (70) was obtained from the American Type Culture Collection (ATCC) and is designated the WT strain in all analyses. Bacterial strains and plasmids are summarized in Table 3, and primers are listed in Table 4. All bacterial strains used in the study were grown in LB broth or on LB agar plates and incubated under aerobic conditions overnight (ON) (16 to 20 h) at 37°C unless otherwise stated. Antibiotic concentrations used for selection purposes were 100 µg/ml ampicillin and 25 µg/ml erythromycin, unless otherwise stated, and were purchased from AMRESCO and Sigma-Aldrich, respectively.

**TABLE 3:** Strains and plasmids used in this study

Strain or plasmid	Genotype or description^*a*^	Reference or source
Strains		
A. baumannii		
ATCC 17978	Noninternational type clone (wild type)	ATCC (70)
Δ*qseBC*	ATCC 17978 with Ery^r^ insertion disruption in *qseBC*	This study
Δ*ygiW*	ATCC 17978 with Ery^r^ insertion disruption in *ygiW*	This study
Δ*hns*	ATCC 17978 with *hns* disrupted by IS*Aba12*	27
Δ*hns*::IS*Aba12*	ATCC 17978 with *hns* disrupted by IS*Aba12*	This study
Δ*qseBC* Δ*hns*::IS*Aba12*	Δ*qseBC* with *hns* disrupted by IS*Aba12*	This study
Δ*ygiW* Δ*hns*::IS*Aba12*	Δ*ygiW* with *hns* disrupted by IS*Aba12*	This study
Δ*ygiW* Δ*hns*::MITE*_Aba12_*	Δ*ygiW* with *hns* disrupted by MITE*_Aba12_*	This study
Δ*hns* pWH0268	Δ*hns* with pWH0268	27
Δ*hns*::IS*Aba12* pWH0268	Δ*hns*::IS*Aba12* with pWH0268	This study
Δ*qseBC* Δ*hns*::IS*Aba12* pWH0268	Δ*qseBC* Δ*hns*::IS*Aba12* with pWH0268	This study
Δ*ygiW* Δ*hns*::IS*Aba12* pWH0268	Δ*ygiW* Δ*hns*::IS*Aba12* with pWH0268	This study
Δ*ygiW* Δ*hns*::MITE*_Aba12_* pWH0268	Δ*ygiW* Δ*hns*::MITE*_Aba12 _*with pWH0268	This study
		
E. coli		
DH5α λ*pir*	F^–^ Φ80*lacZ*ΔM15 Δ(*lacZYA-argF*)*U169 recA1 endA1 hsdR17*(r_K_, m_K_^+^) *phoA supE44* λ^–^ *thi*-*1 gyrA96 relA1* λ*pir,* conjugative strain which can host λ-*pir*-dependent plasmids	87
Plasmids		
pAT04	Tet^r^; pMMB67EH with Rec_Ab_ system	71
pGEM-T Easy	Amp^r^; T-overhang cloning vector	Promega
pVA891	Cml^r^ Ery^r^; Source of Ery^r^ cassette	88
pWH0268	Amp^r^; pWH1266 with *hns* cloned via BamHI restriction site	27

a Abbreviations: Amp, ampicillin; Cml, chloramphenicol; Ery, erythromycin; Tet, tetracycline.

**TABLE 4:** Primers used in this study

Primer function and name	Sequence^*a*^ (5′–3′)	Reference or source
Cloning and sequencing of *hns* genes with integrated mobile genetic elements		
* hns*_F	GAGA**CATATG**ATGCATCATCATCATCATCATATAAATATTAAGAAAATATATTA	27
* hns*_R	TCTC**GGATCC**TTAGATTAAGAAATCTTCAAG	27
M13 F	GTAAAACGACGGCCAG	Promega
M13 R	CAGGAAACAGCTATGAC	Promega
		
Identification of presence of IS		
ACX60_04650_F	CGTATTTGGGTCTTGGGGAA	This study
ACX60_04650_R	CCTTTGGTAAGTACTTTAT	This study
ACX60_18935_F	AGCAACTGAAGCTGAAATTCG	27
ACX60_18935_R	TTGGTTCCGAATTAGACTTGC	27
ACX60_04795_F	CAGTCAGGTTCGCCAT	This study
ACX60_04795_R	GACCAGACAATACAATG	This study
		
Construction of Δ*qseBC* and Δ*ygiW*		
Δ*qseBC*		
Δ*qseBC*_UFR_F	CAATTCCGCGATAAGAGC	This study
Δ*qseBC*_UFR_R	CTATCAACACACTCTTAAGCCTGTTATATCCTGAT	This study
Δ*qseBC*_DFR_F	CGGGAGGAAATAATTCTATTTGCAGTCACAACTGG	This study
Δ*qseBC*_DFR_R	GTAGTAACCAGAACAGCAC	This study
Δ*qseBC*_NOL_F	GGCAAGGACGTCCTGTTT	This study
Δ*qseBC*_NOL_R	GGGCTGAAAAACTTCAAC	This study
Δ*qseBC*_Ery_F	CTTAAGAGTGTGTTGATAG	39
Δ*qseBC*_Ery_R	ATAGAATTATTTCCTCCCG	39
Δ*ygiW*		
Δ*ygiW*_UFR_F	CAGTTGAAATGGCATCCATTAC	This study
Δ*ygiW*_UFR_R	CTCTTAAGGTATAGGAACTTCAAAATTACCCTCTGTTA	This study
Δ*ygiW*_DFR_F	GAGGAAATAAGAAGTTCCTATACTAAATTAATTTCTACATTTATTCC	This study
Δ*ygiW*_DFR_R	GAGA**GCGGCCGC**CTCATTTTAAGTCTCCCATAC	This study
Δ*ygiW*_NOL_F	CGGCATTTATGAGTTTATGCCAG	This study
Δ*ygiW*_NOL_R	GGCTTGCCCCAACTGA	This study
ΔygiW_Ery F	GAAGTTCCTATACCTTAAGAGTGTGTTGATAG	This study
ΔygiW_Ery R	GTATAGGAACTTCTTATTTCCTCCCGTTAAATAATAGATAAC	This study

a Nucleotides in boldface represent incorporated restriction sites: NdeI, CATATG; BamHI, GGATCC; NotI, GCGGCCGC.

### Construction of deletion and complementation derivatives.

A. baumannii ATCC 17978 *qseBC* (ACX60_06100/05) and *ygiW* (ACX60_06095) deletion strains were constructed using the RecET recombinase system (71), with modifications as outlined previously (39). Primers used to generate mutant strains are listed in Table 4. For complementation of insertionally inactivated *hns* genes identified in this study, a previously generated pWH1266 shuttle vector carrying a WT copy of *hns* amplified from A. baumannii ATCC 17978 chromosomal DNA (pWH0268) was used to transform appropriate A. baumannii strains as previously described (27).

### Desiccation survival assay.

Desiccation survival assays followed the method outlined previously (8), with modifications. Briefly, ON cultures were diluted 1:25 in fresh LB broth and grown to late log phase (OD_600_ of 0.8 to 1.0). Cells were subsequently washed three times in sterile distilled water and diluted to an OD_600_ of 0.1. A total of 300 µl was pipetted into the center of individual wells of 6-well culture plates and placed in a laminar-flow hood ON at 25°C to dry. All plates were incubated at 21°C with a relative humidity of 30% ± 2%, maintained by the addition of saturated CaCl_2_ within sealed plastic boxes. Humidity and temperature were monitored over the 30-day time course using a thermohygrometer. CFU were assessed on days 0, 1, 3, 5, 7, 9, 15, 21, and 30. For viable cell quantification, desiccated cells were rehydrated in sterile phosphate-buffered saline, scraped from their respective wells, and serially diluted. Suspensions of diluted cells were plated on LB agar and incubated ON, and desiccation survival was calculated from the number of CFU/ml. Experiments were undertaken in two biological replicates from two independent experiments. Average CFU and standard errors of the means were calculated and graphed.

### Gene cloning and DNA sequencing.

The upstream intergenic and coding regions of *hns* from hypermotile variants obtained after desiccation stress experiments were PCR amplified using Velocity DNA polymerase (Bioline, Australia) with *hns*_F and *hns*_R (Table 4) by following the manufacturer’s instructions. Adenosine treatment was undertaken on purified amplicons prior to T/A ligation with pGEMT Easy (Promega) and transformation into E. coli DH5α λ*pir*. Transformants were screened by PCR, restriction digestion, and DNA sequencing.

### Stability of MITE*_Aba12_* in *hns*.

Five colonies were separately inoculated into 10 ml of LB broth and passaged over a 5-day period using a dilution of 1:10,000. From the fifth passage, a loop of confluent bacterial suspension was streaked onto LB agar and incubated ON. A total of three well-isolated colonies from each of the five biological replicates were randomly selected and PCR screened with *hns*_F and *hns*_R (Table 3) to identify maintenance of the MITE within the *hns* gene.

### Motility assays.

Motility assays for A. baumannii ATCC 17978 WT and mutant derivatives were undertaken as previously described (30). Briefly, a colony was harvested from an LB agar plate grown ON and used to inoculate the center of an LB agar (0.25%) plate. Motility was assessed by visual examination after ON incubation at 37°C. Experiments were performed in duplicate over at least three independent experiments. Images are average representations of results obtained.

### Comparative genomics, alignments, and clustering.

For generation of multiple DNA sequence alignments of all full-length MITE*_Aba12_* copies identified, sequences were obtained from NCBI GenBank and used as input data using Clustal Omega with default parameter settings applied (https://www.ebi.ac.uk/Tools/msa/clustalo/) (34). Prior to alignment, copies of MITE*_Aba12_* identified in the opposite orientation (IRR to IRL) were reverse complemented. In strains with multiple copies of MITE*_Aba12_*, these were numbered (_#1, _#2, _3#, etc.) based on their order from NCBI BLASTN (2.8.0+) outputs (72). Subgroups in the alignment were defined based on the presence of two or more identical MITE*_Aba12_* sequence arrangements, numbered from 1 to 10 and ordered according to abundance. To generate the MITE*_Aba12_*(c) consensus sequence, all elements of 113 bp in length were used as input data and visualized using WebLogo software (35) with default settings applied.

The presence of the composite transposon in M. osloensis CCUG 350 was identified by manual examination of the sequence surrounding the MITE*_Aba12_* element using the genome map tool from the Kyoto Encyclopedia of Genes and Genomes database (http://www.kegg.jp/) (32). To identify this composite transposon in other genomes, nucleotide sequence spanning the gene locus tags AXE82_04585-AXE82_04645 in M. osloensis CCUG 350 was used as a query in BLASTN searches. Sequences from M. osloensis CCUG 350, AXE82_04585-04645 (15,922 bp), and KSH (73), KSH_08645-08655 (7,446 bp), were used to generate a genetic map using the Easyfig 2.2.2 tool (74). To identify the presence of the composite transposon across all sequenced genomes, nucleotide sequences located between the terminal ends of the composite transposon from M. osloensis CCUG 350 (AXE82_04595-AXE82_04625) were used as a query, and comparative BLASTN searches (72) were performed. The alignment between M. osloensis CCUG 350 and A. guillouiae NBRC 110550 was generated using EasyFig 2.2.2 (74) as described above. The composite transposon identified in this study was allocated the name Tn*6645* by the transposon registry (https://transposon.lstmed.ac.uk/tn-registry).

Coding regions, E. coli-derived σ70 consensus promoter sequences, RNA secondary structures, and Rho factor-independent terminators were predicted with MITE*_Aba12_*(c) as the input sequence using NCBI ORF finder (https://www.ncbi.nlm.nih.gov/orffinder/) (31), the Softberry BPROM tool (http://www.softberry.com/berry.phtml?topic=bprom&group=programs&subgroup=gfindb), the RNA Mfold server (http://unafold.rna.albany.edu/?q=mfold/rna-folding-form) (36), and ARNold, a Rho-independent transcription terminator finding tool (http://rna.igmors.u-psud.fr/toolbox/arnold/) (37), respectively. Default settings were applied for all programs mentioned above.

### Characterization of MITE*_Aba12_* TSDs.

A total of 20 bp upstream and downstream of each MITE*_Aba12_* element from BLASTN outputs were used to screen for the presence of TSDs. The AT ratio percentages were calculated based on the number of adenosine or thymidine nucleotides in each of the 9-bp integration sites, and these percentages were plotted against the number of copies harboring each ratio. To identify trends in MITE*_Aba12_* integration sites, all identified TSD sequences were used as input data using WebLogo software (35) with default settings applied.
