# Supplementary figures and images for: MITEAba12, a Novel Mobile Miniature Inverted-Repeat Transposable Element Identified in Acinetobacter baumannii ATCC 17978 and Its Prevalence across the Moraxellaceae Family
Source: mSphere. 2019 Feb 20;4(1):e00028-19. doi: 10.1128/mSphereDirect.00028-19 (PMC6382973; doi:10.1128/mSphereDirect.00028-19)

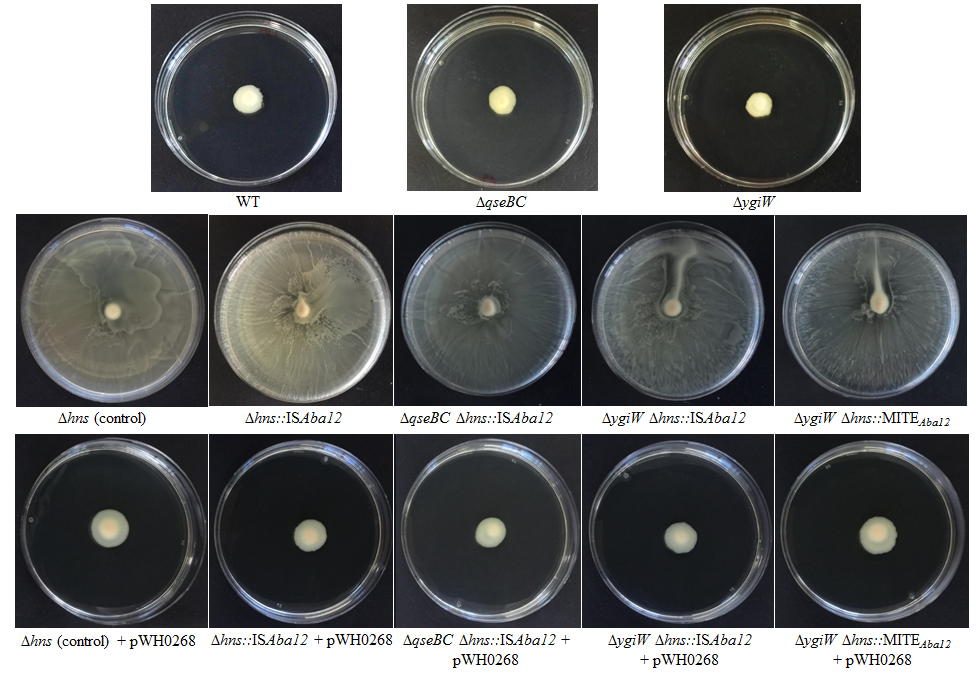

Supplement: FIG S1 [file mSphereDirect.00028-19-sf001.tif]

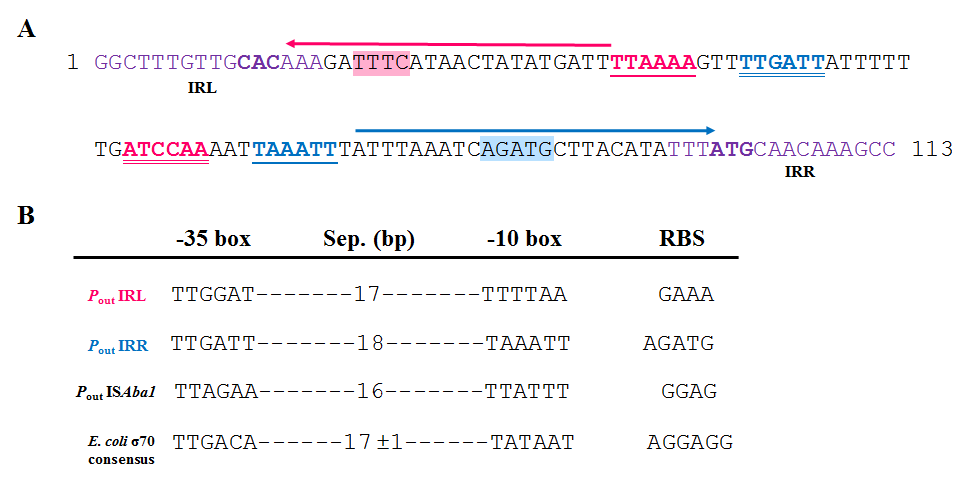

Supplement: FIG S2 [file mSphereDirect.00028-19-sf002.tif]
