# Supplementary material for: MITEAba12, a Novel Mobile Miniature Inverted-Repeat Transposable Element Identified in Acinetobacter baumannii ATCC 17978 and Its Prevalence across the Moraxellaceae Family
Source: mSphere. 2019 Feb 20;4(1):e00028-19. doi: 10.1128/mSphereDirect.00028-19 (PMC6382973; doi:10.1128/mSphereDirect.00028-19)
Supplement: TABLE S1 [file mSphereDirect.00028-19-st001.docx]

**Table S1**

| **Subgroup number** | **Copies of MITE*_Aba12_*** | **Representative MITE*_Aba12_* element** | **Additional MITE*_Aba12_* elements** |
| --- | --- | --- | --- |
| 1 | 27 | *A. baumannii* DS002_#1 | *A. baumannii* DS002_#2-18, 20-22  *A. baumannii* B8300_#1-4  *A. baumannii* B8342_#1  *A. junii* 65_#2 |
| 2 | 17 | *A. baumannii* ATCC 17978 | *A.* *indicus* SGAir0564_#1-3  *A. johnsonii* XBB1_#1-3  *A. schindleri* SGAir, pSGAir0122_#1-2  *A. baumannii* ATCC 17978 ∆*ygiW* ∆*hns*::MITE*_Aba12_*_#1-2  *A. junii* 65_#1  *Acinetobacter* sp. BW3  *M. osloensis* CCUG 350  *A. lwoffii* ED45-23, pALWED2.1  *A. junii* WCHAJ59 *Acinetobacter* sp. ACNIH1_#1 |
| 3 | 5 | *Acinetobacter* sp. DUT-2, unnamed 1 | *Acinetobacter* sp. ACNIH2  *Acinetobacter* sp. ACNIH2, pACI-3569  *A. baumannii* D46, pD46-4  *A. baumannii* A297, pA297-3 |
| 4 | 4 | *A. pittii* WCHAP005046, pOXA58_005046 | *A. pittii* WCHAP100004, pOXA58_100004  *A. pittii* WCHAP005069, pOXA58_005069  *A. baumannii* CHI-32, pNDM-32 |
| 5 | 4 | *A. indicus* SGAir0564_#4 | *A. indicus* SGAir0564_#5-6  *A. baumannii* AR_0083 |
| 6 | 3 | *A. johnsonii* XBB1_#4 | *A. johnsonii* XBB1_#5-6 |
| 7 | 2 | *Acinetobacter* sp. ABNIH28_#3 | *A. baumannii* B8342_#2 |
| 8 | 2 | *A. indicus* SGAir0564_#9 | *A. indicus* SGAir0564_#10 |
| 9 | 2 | *Acinetobacter* sp. SWBY1_#1 | *A. junii* 65_#4 |
| 10 | 2 | *A. johnsonii* XBB1 pXBB1-9 | *A. defluvii* WCHA30 pOXA58_010030 |
